# Supplementary material for: Genetic comparisons of interleukin-17 reveal a framework for complex signaling evolution
Source: bioRxiv. 2026 Apr 14:2026.04.13.718218. Preprint. [Version 1] doi: 10.64898/2026.04.13.718218 (PMC13105070; doi:10.64898/2026.04.13.718218)
Supplement: Supplement 3 [file media-3.zip › Supplemental files for paper/ERC/IL17xIL17_comparisons/IL17RA_IL17RB.pdf]

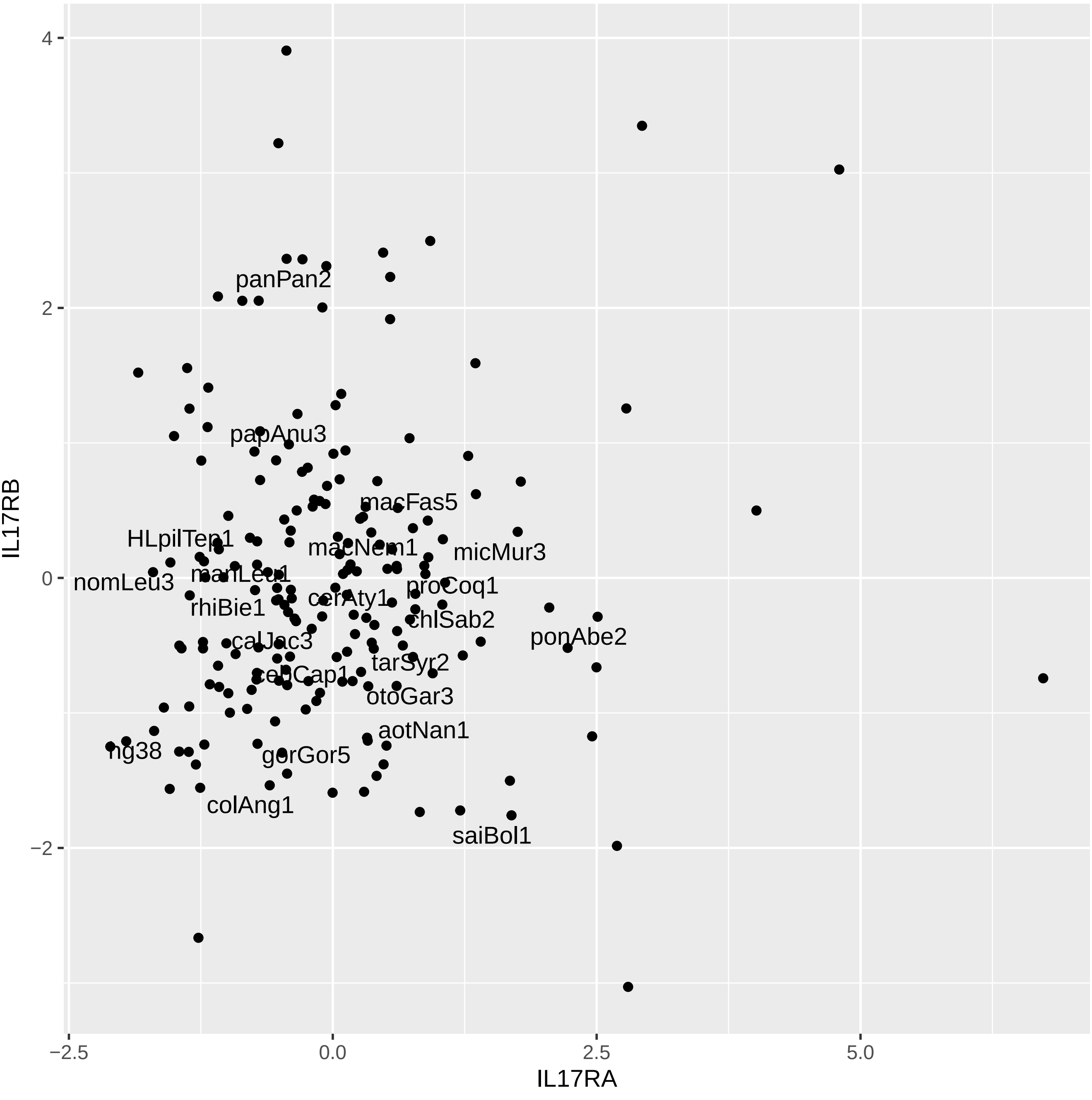

panPan2
papAnu3
macFas5
HLpilTep1
macNem1
micMur3
nomLeu3
manLeu1
proCoq1
cerAty1
rhiBie1
chlSab2
ponAbe2
calJac3
tarSyr2
cebCap1
otoGar3
aotNan1
ng38
gorGor5
colAng1
saiBol1
4
2
0
−2
−2.5
0.0
2.5
5.0
IL17RB
IL17RA
